# Development and Evaluation of Liquid Plaster Loaded with *Chromolaena odorata* Leaf Extract Endowed with Several Beneficial Properties to Wound Healing

**DOI:** 10.3390/gels8020072

**Published:** 2022-01-24

**Authors:** Tanikan Sangnim, Parinya Meeboon, Parinda Phongsewalak, Parichat Prasongdee, Pornsak Sriamornsak, Inderbir Singh, Suwisit Manmuan, Kampanart Huanbutta

**Affiliations:** 1 Faculty of Pharmaceutical Sciences, Burapha University, Chonburi 20131, Thailand; tanikan@go.buu.ac.th (T.S.); 59210176@go.buu.ac.th (P.M.); 59210177@go.buu.ac.th (P.P.); 59210179@go.buu.ac.th (P.P.); suwisit@go.buu.ac.th (S.M.); 2 Department of Pharmaceutical Technology, Faculty of Pharmacy, Silpakorn University, Nakhon Pathom 73000, Thailand; sriamornsak_p@su.ac.th; 3 Academy of Science, The Royal Society of Thailand, Bangkok 10300, Thailand; 4 Chitkara College of Pharmacy, Chitkara University, Patiala 140401, India; inderbir.singh@chitkara.edu.in; 5 School of Pharmacy, Eastern Asia University, Rangsit 12110, Thailand

**Keywords:** *Chromolaena odorata*, film-forming systems, liquid plaster, wound healing, topical dosage forms

## Abstract

Liquid plaster (LP) is a recently developed wound dressing product that can be used to cover wounds in various parts of the body, especially small injuries or wounds in body parts involved in movement. Given the benefits and applications of LP, this study aimed to develop and evaluate *Chromolaena odorata* extract-loaded LP with antimicrobial and hemostasis effects. The study was first conducted through the extraction of *Choromolaena odorata* leaf by using an ethanol maceration technique and identification of the compounds with high-performance liquid chromatography. The LP loaded with *Chromolaena odorata* extract demonstrates an ability to inhibit *S. aureus* and *S. epidermidis* at a MIC of 0.25 mg/mL and MBC of 0.5 mg/mL. The antioxidant activity test was performed by ABTS and DPPH methods demonstrating the free-radical scavenging activity of the extract. The blood clotting activity was established by varying the concentration of *Choromolaena odorata* leaf extract from 0.0625 mg/mL to 1 mg/mL. The formulation of the film-forming system was developed by varying the solvent, polymer, and plasticizer proportions. The optimum formulation displayed fast film-forming with high elasticity of the film. Moreover, the 20 mg/mL herbal extract-loaded LP provided an antibacterial effect with admissible water vapor transmission and low skin irritation. As a result, the study demonstrates the possibility of introducing the *Chromolaena odorata* extract-loaded LP to increase the effectiveness of wound healing and the antibacterial effect on the skin.

## 1. Introduction

Acute skin wounds can be caused from superficial scratches to deep injuries, and they can be completely healed with minimal or no scar formation within 3 weeks [1]. Although most dermal wounds are healed by a natural healing process, this process may be retarded by several factors such as infection, movement, and foreign bodies, etc. [2]. If the wounds are covered with dressing, they are continuously exposed to proteinases and chemotactic, complement, and growth factors, which can be lost in the exposed wound [3]. As a result, various wound dressing materials have been developed and used to protect and keep wounds moist. These dressings also enhance re-epithelialization and collagen synthesis. They also promote angiogenesis by creating hypoxia to the wound bed and decrease wound-bed pH which leads to a decrease in the wound infection. Different types of wound require different properties of wound-dressing products. For example, burn injuries necessitate wound dressings that are gentle on the patient’s skin. The proper dressing also provides antimicrobial activity and maintains a moist environment [4]. Nonetheless, there are still some wounds for which there are no appropriate wound dressings available. For instance, small wounds in the area of the nail nook or crook of the finger are not suitable to treat with ordinary wound plaster. Consequently, a film-forming system concept has been applied to fabricate liquid plaster products for small wound covering [5]. For the last 5 years, film-forming systems for wound healing application or liquid plaster products have received interest in research and commercial product development due to the fact that they provide various benefits over conventional plasters, such as being suitable for curved skin surfaces [6], being pain free during patch removal, causing low skin irritation [7], and being neat and clean (thanks to their transparent film) [8].

The film-forming system is a new topical and transdermal formulation that starts off as a liquid and then forms a film in situ, or after being applied to the skin. These systems are mainly composed of an active ingredient and a film-forming agent dissolved in a vehicle which evaporates on the skin and leaves behind a film of excipients along with the drug. Numerous film-forming excipients together with suitable solvents were utilized in the film-forming system such as polyvinyl alcohol (PVA) [9], ethyl cellulose, Eudragit RS 100 [10], and polyvinyl pyrrolidone [11]. In this study, Plastoid^®^ B (Poly (butylmethacrylat-co-methylmethacrylate)) was applied as a film-forming agent due to its ability to provide highly flexible films with a transparent color [12]. Therefore, Plastoid^®^ B has previously been used as a film-forming agent in various transdermal patch formulations [13]. However, it has not yet been evolved into a film-forming technology due to Plastoid^®^ B is still novel excipient for topical formulation. Plastoid^®^ B is soluble in several solvents such as esters, ketones, alcohol, glycol ethers, and aromatics which can evaporates abruptly after spreading on the skin. This makes the possibility of using Plastoid^®^ B as an ideal film-former for the film-forming system.

For more than 5000 years, several plant extracts and natural drugs such as propolis [5], liquid neem (*Azadirachta indica*) extracts [14], *Calendula officinalis* [15], *Chamomilla recutita* [16], and *Centella asiatica* have been used in wound care to enhance the healing process [17]. These herbal remedies perform disinfection, debridement, and offer a conducive environment in which to promote the natural healing process [17]. In this work, an extract from *Chromolaena odorata* leaves was used and loaded to the liquid film plaster for the acceleration of the wound healing process. *Chromolaena odorata* is in the family of Asteraceae. Aqueous extracts and decoctions from Chromolaena leaves have been used traditionally throughout Thailand and Vietnam in the treatment of soft tissue and burn wounds [18].

This liquid extract improves hemostatic activity, inhibits wound contraction, stimulates the granulation tissue and re-epithelialization processes and can, therefore, aid wound healing, minimize post-burn scar contracture, as well as deformities [19]. *Chromolaena odorata*, on the other hand, has yet to be transformed into contemporary dosage forms. This implies that the usage of this potentially wound-healing plant would be restricted.

The aim of this study was to develop liquid plaster loaded *Chromolaena odorata* leaf extract for wound healing purposes. *Choromolaena odorata* leaves were extracted by ethanol maceration. Then, the obtained extract was identified by HPLC technique. After that, antimicrobial, anti-inflammatory, antioxidant, blood clotting test activities, and cytotoxicity of the extract were evaluated. In the meantime, liquid plaster was formulated using Plastoid^®^ B as a film forming agent then the proper concentration of *Chromolaena odorata* leaf extract was added to the optimized liquid plaster formulation. Finally, drying time, mechanical properties, water vapor transmission rate, and antimicrobial activities of film loaded active ingredient was tested and reported.

## 2. Results

### 2.1. Physical Appearance, Identification and Quantification of Active Substance from Chromolaena odorata Extracts

**Figure 1:**
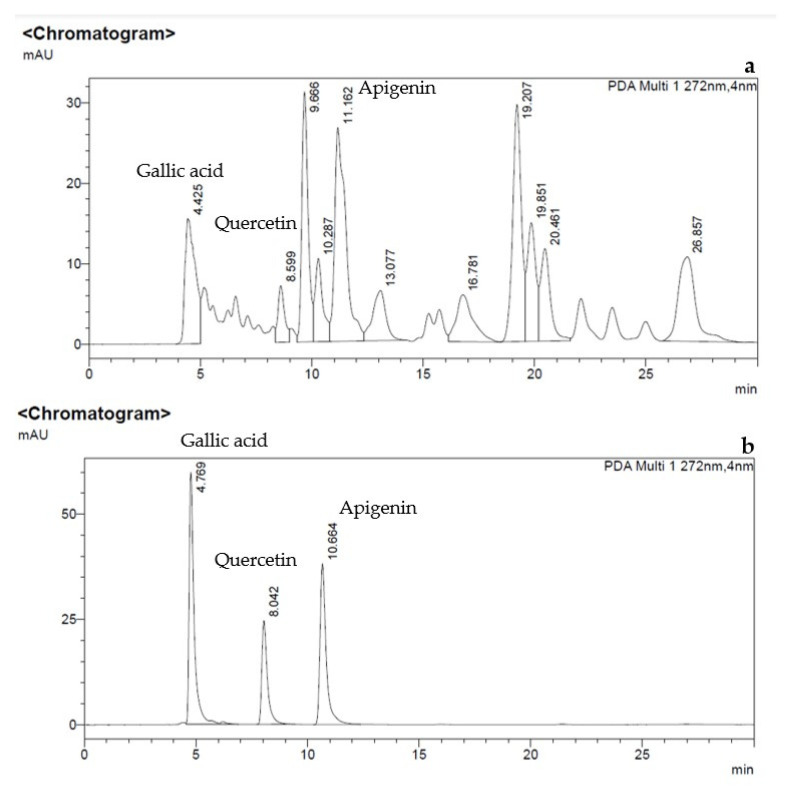
HPLC chromatograms of (**a**) *Chromolaena odorata* extract and (**b**) the reference standards, gallic acid, quercetin, and apigenin.

The crude product after evaporation of *Chromolaena odorata* ethanol extracts was dark green slurry and its extraction yield (% of the final extract weight in comparison to the crude weight) was 11.67 ± 0.99%. After that, the major compounds from the *Chromolaena odorata* extract were identified and quantified by HPLC technique as shown in Figure 1a. The HPLC chromatograms of the reference standards, gallic acid, quercetin, and apigenin provided retention time peaks of 4.7, 8.0, and 10.6 min, respectively (Figure 1b). The retention times are in agreement with the previous study by Chen and team which reported the following HPLC conditions: the mobile phase composition of methanol/acetonitrile/acetic acid/phosphoric acid/water at a 200/100/10/10/200 ratio, detecting the wavelength of 352 nm, and retention time of gallic acid, quercetin and apigenin were 4.2, 7–9 and 12–15 min, respectively [20]. After the standard curves were generated, the amount and percent yield of gallic acid, quercetin, and apigenin from *Chromolaena odorata* extracts were quantified as presented in Table 1. The amount of the flavonoids from crude was close to the previous work by Thophon and colleagues reporting that gallic acid and quercetin from *Chromolaena odorata* ethanol extracts were 0.154 g and 0.031 g per 100 g of the crude extract, respectively. Moreover, the other flavonoid substances were found in the amount of 1.016 g from 100 g of the extract [21]. These findings support the preservation of the major active chemicals found in *Chromolaena odorata* leaves collected in Chonburi province. Additionally, as previously documented, ethanol maceration proved a suitable extraction technique for flavonoid extractions [22].

### 2.2. Antimicrobial Activity of Chromolaena odorata Extracts

The MIC of *Chromolaena odorata* extracts was determined by broth dilution, then MBC was measured by subculturing the broths used for MIC determination onto fresh agar plates. Finally, the antimicrobial activity of *Chromolaena odorata* extracts at different concentrations was confirmed by the agar well diffusion method. As presented in Table 2 MIC of *Chromolaena odorata* extracts against *S. epidermidis* and *S. aureus* were equivalence at 0.25 mg/mL and those of MBC were 0.5 mg/mL. The MIC and MBC results from this study were better than previously reported by Thophon and team [21] who informed us that the MIC of ethanolic leaf of *Chromolaena odorata* extracts inhibiting *S. epidermidis* and *S. aureus* were 0.81 and 6.25 mg/mL, respectively. The MBC against *S. epidermidis* and *S. aureus* was 1.62 and 12.5 mg/mL, respectively.

**Figure 2:**
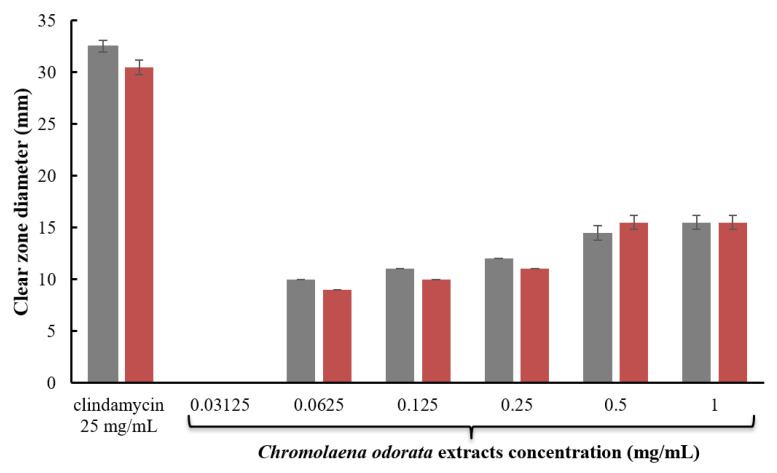
Clear zone diameter of 25 mg/mL clindamycin solution and different concentrations of *Chromolaena odorata* extracts against (gray) *S. epidermidis* and (red) *S. aureus.*

Antimicrobial activity of different concentrations *Chromolaena odorata* extracts and 25 mg/mL clindamycin solution was further tested by the agar-well diffusion method as illustrated in Figure 2. When the concentration of *Chromolaena odorata* extracts was increased, the antibacterial activity against *S. epidermidis* and *S. aureus* increased. Although the inhibition zone of 1 mg/mL *Chromolaena odorata* extracts was lower than that of 25 mg/mL clindamycin solution (commercial product concentration), the tested concentration of *Chromolaena odorata* extracts was lower than the clindamycin solution by 25 times. The results are comparable to those reported by Thophon et al., who found that *Chromolaena odorata* extracts had inhibition zones of 16.0 ± 0.50 and 13.0 ± 1.0 against *S. epidermidis* and *S. aureus*, respectively [21].

### 2.3. Antioxidant Activity of Chromolaena odorata Extracts

IC_50_ (half maximal inhibitory concentration) values of *Chromolaena odorata* extracts tested by ABTS and DPPH assay were 88.31 ± 4.42 and 143.92 ± 3.40 µg/mL, respectively. The standard references for these tests were gallic acid which is a strong antioxidant with free radical scavenging activities. In this study, gallic acid (positive control) showed antioxidant activity at IC_50_ of 1.24 ± 0.0996 and 1.97 ± 0.14 µg/mL for ABTS and DPPH assay, respectively, which was lower than the extract. The scavenging activity result from ABTS test of *Chromolaena odorata* extracts exhibited lower IC_50_ than that of DPPH method. The antioxidant activity result of the *Chromolaena odorata extracts* was in agreement with the previous report by Omokhua and colleagues [23].

### 2.4. Blood Coagulation Test of Chromolaena odorata Extract

**Figure 3:**
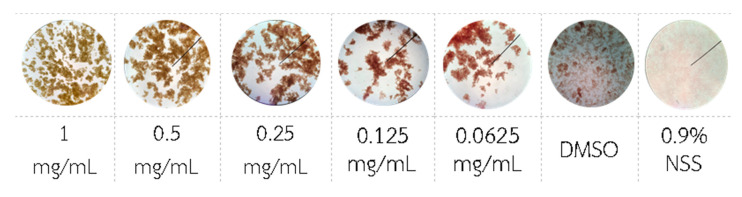
Blood coagulation test of *Chromolaena odorata* extract (0.0625–1mg/mL), DMSO, and NSS.

Blood coagulation test of *Chromolaena odorata* extracts, positive (DMSO) and negative (normal saline) controls were depicted in Figure 3. It was found that *Chromolaena odorata* extracts at the concentration range of 0.0625–1 mg/mL provided platelet coagulant activities with an average platelet coagulation size of 0.5–1.0 mm. Protein precipitated in the positive control (DMSO), producing blood coagulation. Normal saline, on the other hand, had no effect on blood coagulation. The blood coagulation mechanism of *Chromolaena odorata* extracts was proposed before by Akomas et al. explaining that the hemostatic property of *Chromolaena odorata* has been attributed to the presence of tannins and saponins in the leaf extracts. Tannins have been implicated in the hemostatic activity of plants where they arrest bleeding from damaged or injured vessels by precipitating proteins to form vascular plugs. Moreover, saponins have been reported to have precipitating and coagulative properties [24].

### 2.5. Cytotoxicity

**Figure 4:**
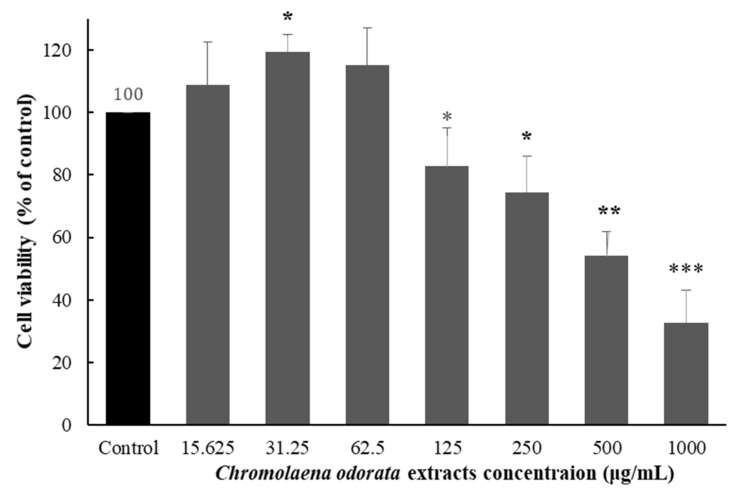
Effects of *Chromolaena odorata* extract on MRC-5 cell viability. Cells were exposed to 15.625–1000 µg/mL of *Chromolaena odorata* extract for 24 h. The percentage of cell viability was determined by MTT cell viability assay. The results were represented as a percentage of control. Data were expressed as the mean ± SEM (*n* = 3). *** *p* < 0.001, significantly different from the control, ** *p* < 0.01, significantly different from the control, * *p* < 0.05, significantly different from the control.

Effects of *Chromolaena odorata* extract on MRC-5 cell viability was monitored by the MTT cell viability assay. The cells were treated by 15.625–1000 µg/mL of *Chromolaena odorata* extract for 24 h. As illustrated in Figure 4, at 125 μg/mL and higher concentrations of *Chromolaena odorata* extract, cell viability was severely reduced. The IC_50_ of *Chromolaena odorata* extract cytotoxicity was 673.23 μg/mL which is moderate toxicity for natural component [25]. This IC_50_ value was slightly higher than the previous report by R.N. Asomugha and colleagues revealing that the cytotoxicity IC_50_ of *Chromolaena odorata* extract to brain shrimp was 324–392 μg/mL [26]. This might be owing to the fact that different test cells and extraction methods were used.

### 2.6. Formulation of Liquid Plaster

Plastoid^®^ B could be clearly dissolved in ethyl acetate during the concentration range of 5–35% (*w*/*w*) (F1.1–1.7 from Table 4). However, only the dissolved Plastoid^®^ B at the concentration range of 25–35% (*w*/*w*) provided transparent thin film after drying.

#### 2.6.1. Rheological Properties

**Figure 5:**
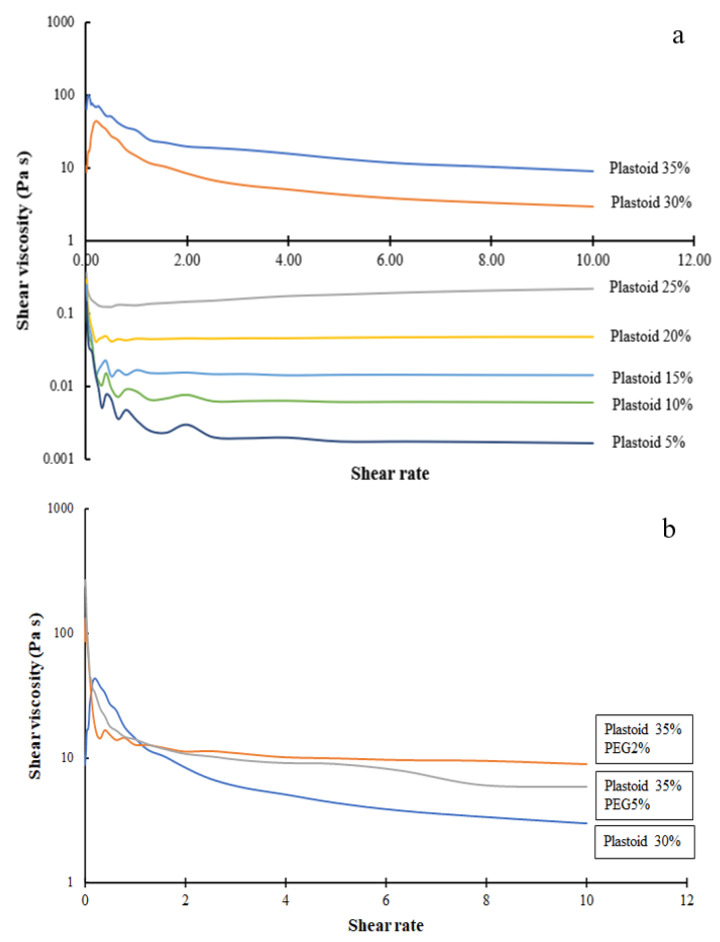
Flow curves of (**a**) different concentration Plastoid^®^ B and (**b**) different added PEG in 30% Plastoid^®^ B solution.

The rheological flow curves of different formulation liquid plasters are shown in Figure 5a. The dissolved Plastoid^®^ B at all concentrations showed shear thinning, a non-Newtonian characteristic in which viscosity reduces as shear rate increases, as seen in the figure [27]. However, yield stresses exist for Plastoid^®^ B gels with a concentration of 30% and 35% [28]. As shown in Figure 5b, adding PEG enhanced shear viscosity but the rheology behavior still was shear thinning. This finding is in line with a recent study that found PEG decreases the degree of polymer chain tangling. As a result, the mobility of the molecular chains improves, making it simpler for them to move under shear stress [29].

**Figure 6:**
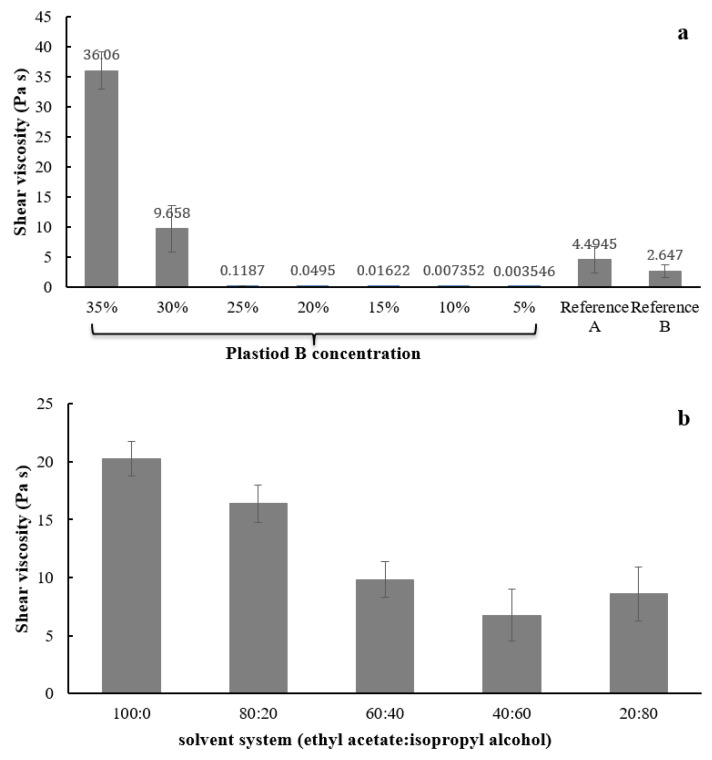
Shear viscosity of 30% Plastoid^®^ B solution at (**a**) different polymer concentrations and (**b**) solvent systems (ethyl acetate:isopropyl alcohol).

The shear viscosity of various Plastoid^®^ B at shear rate of 1 s^−1^ (shear rate simulating pouring action [30]) is illustrated in Figure 6. As shown in Figure 5a, the viscosity was dependent on the polymer concentration (30–35% Plastoid^®^ B). Plastoid^®^ B shear viscosities were not different at concentrations ranging from 5 to 25%. The shear viscosity of reference product A and B were 2.64 ± 0.07 Pa s and 4.49 ± 1.38 Pa s, respectively, which was close to that of 30% Plastoid^®^ B (9.66 ± 5.18 Pa s). Therefore, formulation of 30% Plastoid^®^ B was selected for further testing and product development. Then, different solvent systems (ethyl acetate:isopropyl alcohol) were applied to dissolve Plastoid^®^ B at a concentration of 30% and their shear viscosities were measured as depicted in Figure 6b. The viscosity of all dissolved Plastoid^®^ B preparations was considerably decreased when the isopropyl alcohol component was increased. When the isopropyl alcohol portions were 40 or 60% of the solvent, the shear viscosities were not different and close to those of the commercial products.

#### 2.6.2. Liquid Drying Time

**Figure 7:**
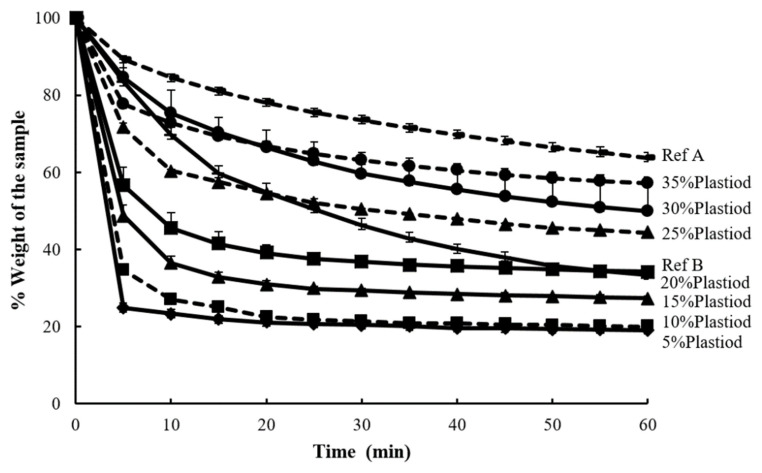
Drying time profiles of Plastoid^®^ B at different concentrations (5–35%) and the commercial reference products.

Drying time profile of Plastoid^®^ B solution at different concentrations (5–35%) and the commercial products are presented in Figure 7. The weight dramatically decreased at first 5 min especially those of 5–20% Plastoid^®^ B solutions. The reason for this is that formulations with low polymer concentrations have highly volatile solvents, which cause rapid evaporation. Furthermore, the Plastoid^®^ B solution concentration of 25–35% produced a drying time profile that was comparable to the reference products.

**Figure 8:**
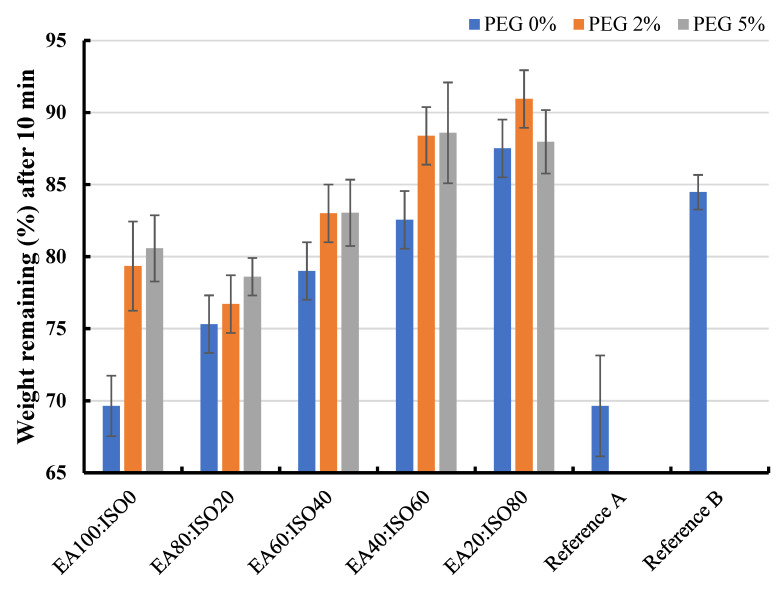
Weight remaining percentages of the 30% Plastoid^®^ B solution with different solvent systems (EA (ethyl acetate): ISO (isopropyl alcohol)) and PEG-added portions comparing to the commercial reference products.

The effect of solvent systems (ethyl acetate: isopropyl alcohol) and PEG portion (0–5%) on drying time were also monitored by W_10min_ value which is the weight of formulation after 10 min of the time to dry test. The W_10min_ values of different Plastoid^®^ B formulations are shown in Figure 8. By increasing the amount of isopropyl alcohol in the formulation, the rate of solvent evaporation was slowed. This is due to the boiling point of ethyl acetate (77.1 °C) being lower than that of isopropyl alcohol (82.5 °C) [31]. Furthermore, the majority of the results show that PEG slowed solvent evaporation, resulting in a higher weight remaining in the formulation. This outcome obeys Raoult’s law, explaining that if any non-electrolyte substances (in this case, PEG) are dissolved in any solvent, the vapor pressure above the solution will be less than the pure solvent [32]. However, the W_10min_ values of all the Plastoid^®^ B solutions were about the same as commercial products (69.23–84.10%).

#### 2.6.3. Film Mechanical Properties

**Figure 9:**
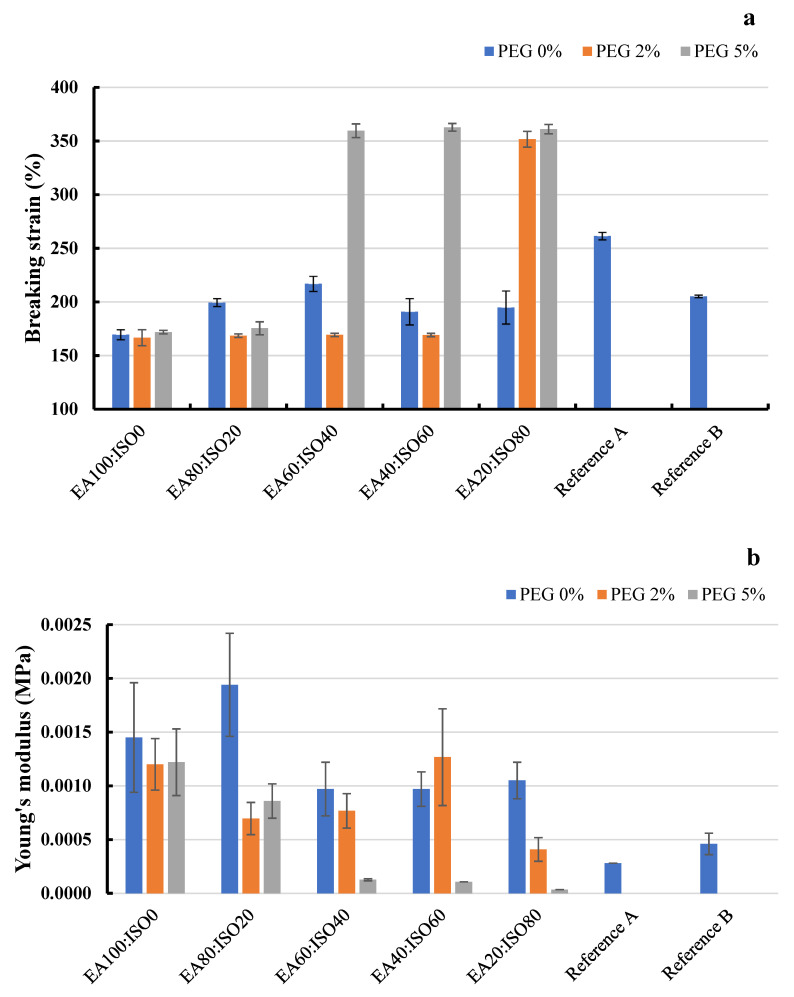
(**a**) Breaking strain and (**b**) Young’s modulus of the films prepared from 30% Plastoid^®^ B solution with different solvent systems (EA (ethyl acetate): ISO (isopropyl alcohol)) and PEG added portions comparing to the commercial products.

Breaking strain and Young’s modulus of the films prepared from 30% Plastoid^®^ B solution with different solvent systems (ethyl acetate, isopropyl alcohol) and PEG portions compared to the commercial reference products are shown in Figure 9. It was found that adding PEG could enhance film elongation. Moreover, the maximum breaking strain was around 350%, which was greater than that of the reference products (Figure 9a). The Young’s modulus values were in consonance with the breaking strain values. As shown in Figure 9b, the Young’s modulus values were reduced when PEG was added to the liquid plaster formulation. PEG works as a plasticizer well in a solvent ratio (ethyl acetate: isopropyl alcohol) range of 60:40 to 20:80. This might be because of the polarity difference between ethyl acetate (relative polarity of 0.228) and isopropyl alcohol (relative polarity of 0.546) [33]. As reported in a previous research study [34], the higher portion of isopropyl alcohol elevated the solvent polarity, which can escalate film elongation. This might be due to the fact that PEG 400 is a hydrophilic molecule with a dielectric constant of 12.4, allowing it to distribute completely across polymer chains in a high polarity solvent [35].

### 2.7. Physical Properties and Antimicrobial Activity of Liquid Plaster Formulation Loaded Chromolaena odorata Extract

The liquid plaster formulation composed of 30% Plastoid^®^ B, 2% PEG, and the solvent of ethyl acetate and isopropyl alcohol at a ratio of 60:40 was selected for *Chromolaena odorata* extract loading because this formulation offered proper viscosity, drying period, and film mechanical properties. The *Chromolaena odorata* extract loading concentration of 20 mg/mL was used for the formulation because this concentration provided antimicrobial activity, antioxidant activity, and hemostatic property with low cytotoxicity. The prepared liquid plaster formulation loaded with *Chromolaena odorata* extract was evaluated under the topics of shear viscosity, time to dry, Young’s modulus, water vapor transmission, and antimicrobial activity to monitor the effect of the herbal extract on formulation properties.

#### 2.7.1. Shear Viscosity, Film Drying, and Young’s Modulus

The shear viscosity, film drying in terms of W_10min_, and Young’s modulus of the blank liquid plaster formulation and liquid plaster formulation loaded with *Chromolaena odorata* extract compared to the reference products are presented in Table 3. Shear viscosity and film drying time were not affected by adding *Chromolaena odorata* extract to the formulation. The extract elevated Young’s modulus from the blank film, indicating the liquid plaster formulation loaded with *Chromolaena odorata* extract has higher strength with similar film stretching to the blank film. The Young’s modulus values of the liquid plaster containing *Chromolaena odorata* and the referred products, on the other hand, are acceptable since they can still be stretched. The shear viscosity at 1 s^−1^ of the liquid plaster formulations (extract load/blank) was higher than the reference products. The liquid plaster formulation’s film drying time was quite similar to that of the reference product A.

#### 2.7.2. Water Vapor Transmission

**Figure 10:**
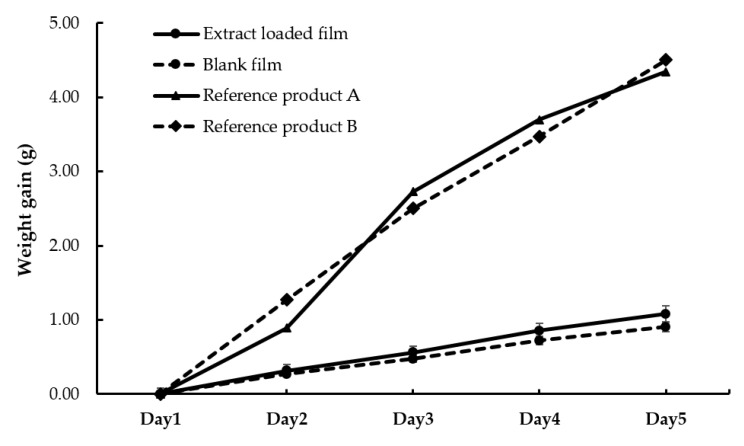
Water vapor transmission study of the *Chromolaena odorata* extract loaded cast film and blank film.

The water vapor transmission study of the *Chromolaena odorata* extract loaded cast film and blank film was presented in Figure 10. The experiment lasted 5 days, with the weight of the calcium chloride-filled container covered with films steadily increasing. The weight gain of the bottle covered with the *Chromolaena odorata* extract-loaded cast film was significantly higher than that of blank film. The water vapor transmission rate of the *Chromolaena odorata* extract-loaded cast film and blank film were 110.13 and 91.96 g m^−2^ d^−1^, respectively. The water vapor transmission rates of the reference products A and B were higher than those of the extract-loaded film and blank film, which were 442.35 and 458.66 g m^−2^ d^−1^, respectively.

Although the prepared liquid plaster film had a lower water vapor transmission rate than the reference products, the values were nevertheless comparable to the commercial wound dressing products (50 to 6512 g m^−2^ d^−1^) described in the previous study [36]. As a result, the herbal extract-loaded films appear to allow rather more water vapor to pass through than the blank film. This was explained before by Saringat and his research team, reporting that incorporation of an insoluble substance into the film caused irregular spots and holes enhancing moisture permeability [37].

#### 2.7.3. Antimicrobial Activity by Agar Well Diffusion Method

**Figure 11:**
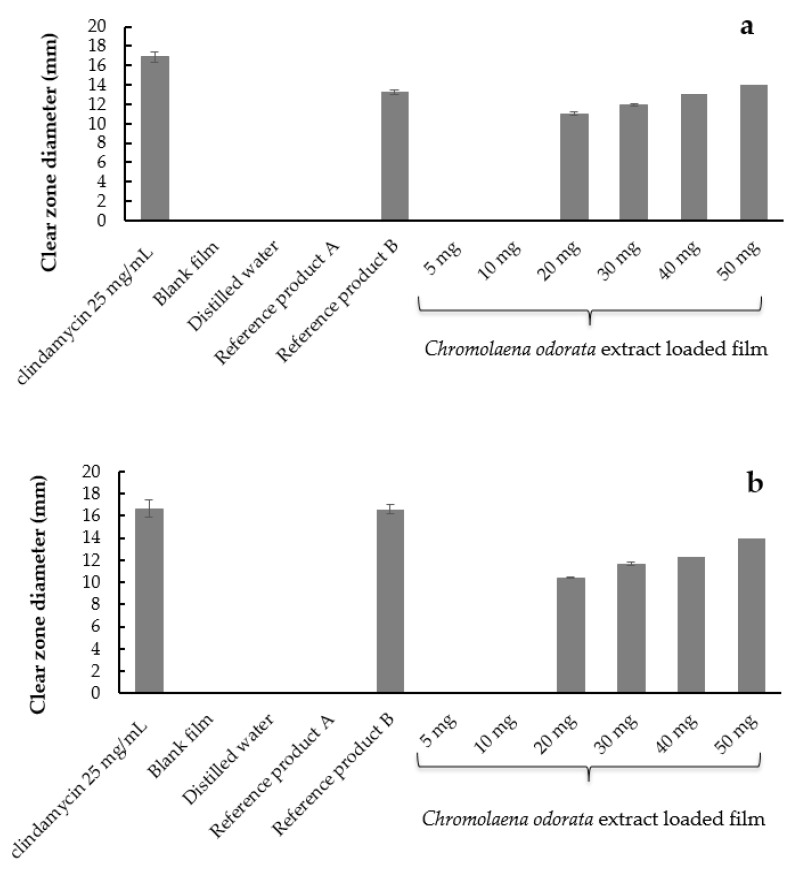
Clear zone diameter of 25 mg/mL clindamycin solution, blank film, distilled water, the reference products A and B, and different concentrations of *Chromolaena odorata* extract-loaded film against (**a**) *S. epidermidis* and (**b**) *S. aureus*.

The clear zone diameter of the 25 mg/mL clindamycin solution (positive control), negative controls, the reference products, and different concentrations of *Chromolaena odorata* extract loaded film against *S. epidermidis* and *S. aureus* are illustrated in Figure 11. The antimicrobial activity of the *Chromolaena odorata* extract-loaded liquid plasters against *S. epidermidis* and *S. aureus* by the agar-well diffusion method was in the same direction. The liquid plasters containing 20–50 mg (4X–10X MBC) *Chromolaena odorata* extract inhibit growth of *S. epidermidis* and *S. aureus*. While the clear zone could not be observed from the negative controls (blank films and distilled water) and the liquid plasters containing 5–10 mg of *Chromolaena odorata* extract, they could be observed from the negative controls (blank films and distilled water). The reason that the liquid plaster film containing a low concentration of *Chromolaena odorata* extract could not inhibit microbial growth was explained in the work of Rosa and team [38]. The result was discussed, and it was found that films require an adequate concentration and dispersion of the antimicrobial compound in their surface matrix. Once the concentration of the antimicrobial compound is sufficient, the compound will be released from all the points on the surface, reaching the minimum bactericidal concentration.

### 2.8. In Vivo Study of Skin Irritation, Transepidermal Water Loss and Skin Density after Liquid Plaster Use

**Figure 12:**
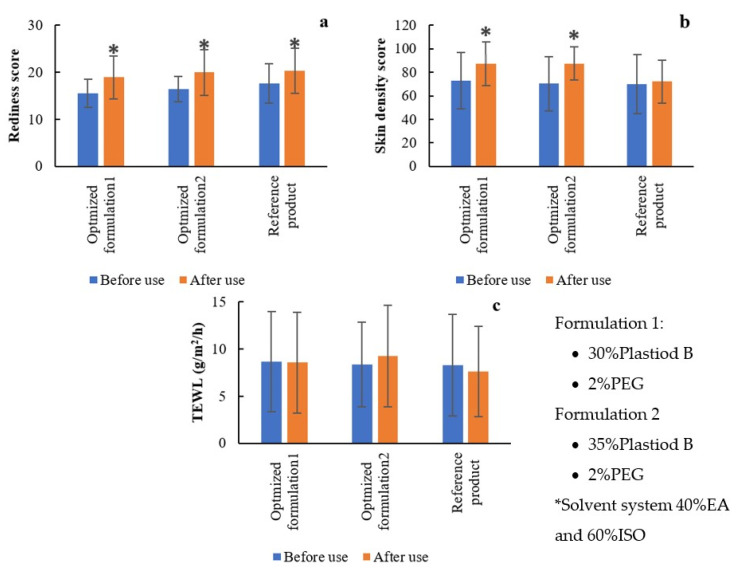
(**a**) Redness score, (**b**) skin density, and (**c**) transepidermal water loss before and after liquid plaster use. * *p* < 0.05, significantly different from the before use.

Skin irritation, transepidermal water loss, and skin density of 27 healthy volunteers after use of liquid plaster and the reference products were measured using Antera 3D (Miravex, Ireland) and DermaLab Combo (Cortex, Denmark). Two optimized liquid plaster formulations (Formulation 1: 30% Plastiod B, 2% PEG and Formulation 2: 35% Plastiod B, 2% PEG under solvent system of 40% ethyl acetate: 60% isopropyl alcohol) and the reference product were selected for the in vivo evaluation as depicted in Figure 11. After usage, all of the products slightly induced skin redness (Figure 12a). The images taken from the Antera 3D is shown in Figure S1. They were not, however, the source of the severe skin irritation and rash. More redness scores may result from film peeling off, which is common in adhesive plaster application, and the redness disappeared within 5–10 min [39]. As demonstrated in Figure 12b, after using the herb extract-loaded optimization liquid plaster, skin density slightly rose as measured by a high frequency ultrasound probe [40]. This might be because the solvents (ethyl acetate and isopropyl alcohol) used in the liquid plaster altered epithelial tissue resulting in higher skin density, temporarily [41]. All liquid plaster samples had no effect on skin integrity, therefore the amount of water that passively evaporates through skin to the external environment did not alter as seen in Figure 12c.

## 3. Conclusions

In the present study, *Chromolaena odorata* leaves were extracted by the maceration method with ethanol. By the HPLC method monitoring, the main active compounds (gallic acid, quercetin, and apigenin) were present in the extract. The extract demonstrated several pharmacological activities, including antibacterial, antioxidant, and blood coagulation, which can enhance the wound-healing process. The cytotoxicity test of the extract exhibited moderate toxicity for natural components. The liquid plaster formulation was optimized by varying the plastoid^®^ B and PEG concentrations dissolved in several solvent systems. The formulation of 30% plastoid^®^ B, 2% PEG, and a solvent mixture of 60:40 ethyl acetate and isopropyl alcohol had shear viscosity, drying time, and film mechanical characteristics that were appropriate for liquid plaster dosage forms and comparable to the reference products. The *Chromolaena odorata* extract could successfully load the liquid plaster and, at a concentration of 20–50 mg/mL, the plaster could inhibit growth of *S. epidermidis* and *S. aureus*. The dry film allowed water vapor transmission at a rate of 110.13 g m^−2^ d^−1^. The liquid plaster containing *Chromolaena odorata* extract did not produce significant skin irritation in the in-vivo skin test. Furthermore, the finished product could improve skin density. According to the findings, the *Chromolaena odorata*-loaded liquid plaster has a significant chance of being used in clinical settings to treat infected wounds in people and animals. To establish the test results in this study, the true scenario of the product utilized in the wound healing study must be investigated further.

## 4. Materials and Methods

### 4.1. Materials

Plastoid^®^ B (Lot No. G171110026) was kindly donated by Evonik industries, Germany. *Chromolaena odorata* leaves were collected in Chonburi province in Thailand. Ethanol (Lot No. 20060068) was received from Labscan. Ethyl acetate (Lot No. 1909051114) was from Kemaus, Australia. Isopropyl alcohol (Lot No. G201102) and polyethylene glycol 400 (Lot No. 00400010076736) were obtained from Krungthepchemi Co., Ltd., Thailand. Standard substances of gallic acid (Lot No. AO405480), apigenin (Lot No. BCBZ4566) and quercetin (Lot No. STBH0486) were purchased from Sigma Aldrich, USA. All other chemicals were of reagent grade and used without further purification.

### 4.2. Chromolaena odorata Extract

The fresh leaves of *Chromolaena odorata* were collected in June 2020 in Chonburi province area and then cleaned with water. The *Chromolaena odorata* leaves were identified by Dr. Boonyadist Vongsak, Faculty of Pharmaceutical Sciences, Burapha University, Thailand. The voucher specimens (KM No.0415001, KM No.0415002, and KM No.0415003) were deposited at the Faculty of Pharmaceutical Sciences, Burapha University, Thailand. After that, 20 g of fresh leaves were soaked with 200 mL of 95% (*v*/*v*) ethanol for 24 h. The mixtures were filtered through Whatman filter paper No.1, and concentrated using a rotary evaporator (R-100, Buchi, Switzerland) at 78 °C. The dark brownish-green viscous residues were dried by freeze-drying (freeze dryer, Labconco, Edwards Limited, UK) for 24 h. The dried product was kept in a tightly closed brown vial at 4 °C until used. Dimethyl sulfoxide (DMSO) at a concentration of 99.9% was used as a dissolving agent to make further tests.

### 4.3. Identification and Quantification of Active Substance from Chromolaena odorata Extracts

According to the previous reports [42], three important active substances (gallic acid, quercetin, and apigenin) of *Chromolaena odorata* extracts can be used to monitor the efficiency of the extraction process. The isolated flavonoids (gallic acid, quercetin, and apigenin) were quantified using high-performance liquid chromatography coupled with an ultraviolet detector (HPLC-UV) (SPD-M20A, Shimadzu, Japan), as previously reported and validated by Xiao-qing Chen and Jian-bo Xiao [20]. The analytical column used in the present study was a C18 reverse-phase KinetexTM column (C18, 2504.6 mm, pore size 5 m, Phenomenex, CA, USA). The mobile phase was a mixture of methanol, acetonitrile, acetic acid, phosphoric acid and water (200:100:10:10:200 by volume). The detecting wavelength was 272 nm and the flow rate was 0.60 mL/min. The volume of the injecting sample was 6.0 l. The HPLC system was set to run at room temperature (28 ± 1 °C).

The referenced standard solutions of gallic acid, quercetin, and apigenin were prepared by dissolving in methanol in the concentration range of 1.5625–100 µg/mL. Then the prepared standard solutions were filtered through a 0.45 m membrane before being injected into the HPLC. After that, the standard curves of gallic acid, quercetin, and apigenin were generated. The *Chromolaena odorata* extracts sample was prepared by dissolving the crude extracts in methanol at the concentration of 1 mg/mL. Then the sample was filtered by a 0.45 µm membrane before being injected into the HPLC with similar conditions as described before.

### 4.4. Antimicrobial Activity of Chromolaena odorata Extracts

*Staphylococcus aureus* (ATCC6538) and *Staphylococcus epidermidis* (ATCC14990) strains were selected as testing organisms due to their representation of Gram-positive bacteria on human skin and mucosal surfaces [43]. The bacterial stock cultures were incubated at 37 °C for 24 h on nutrient agar. The bacterial suspensions were compared to a 0.5 McFarland turbidity standard to obtain 10^7^–10^8^ CFU/mL.

#### 4.4.1. Disk Diffusion Method

The antimicrobial activity of *Chromolaena odorata* extracts against two pathogenic bacteria was investigated by the agar disk diffusion method [5]. The herbal extracts with concentrations of 100, 200, and 300 mg/mL were screened for antibacterial activity against *S. aureus* and *S. epidermidis*. The test cultures were spread on the nutrient agar plate. Whatman filter paper (No.1) as a disk was cut 0.6 cm in diameter, adsorbed with *Chromolaena odorata* extract solutions and then placed on a prepared plate. Plates were incubated at 37 °C for 18 to 24 h. DMSO was used as a negative control. The zones of growth inhibition (including the diameter of the disk) were recorded in diameter (mm). The experiments were conducted in triplicate.

#### 4.4.2. Minimum Inhibitory Concentration (MIC)

The MIC of *Chromolaena odorata* extract was established using the macro broth dilution procedure to estimate the liquid plaster loading dose of the herbal extract. The testing microbes were prepared by transferring Staphylococcus aureus and Staphylococcus epidermidis to Mueller Hinton Broth (MHB) and incubated at 37 °C for 3–4 h. To determine the MIC, the *Chromolaena odorata* extract was diluted in test tubes to concentrations of 1000, 500, 250, 125, 62.5, and 31.25 μg/mL. Then, in each test tube containing *Chromolaena odorata* extract, 5 mL of MHB was added. The *Chromolaena odorata* extracts and microbe mixes were then incubated for 16–18 h at 37 ° C. The turbidity of the mixture in the test tubes after incubation was used to monitor the growth of microorganisms. The MIC was determined to be the lowest concentration of propolis extract that can inhibit microbial growth [44].

#### 4.4.3. Minimum Bactericidal Concentration (MBC)

The MBC of *Chromolaena odorata* extract was explored by streaking the turbid mixture from the MIC test on the surface of Mueller Hinton Agar (MHA) and incubating at 37 °C for 16–18 h. If the concentration of *Chromolaena odorata* extract is high enough, microbial colonies will not be found on the MHA plate [45].

### 4.5. Antioxidant Activity of Chromolaena odorata Extracts

Two radical scavenging assays using 2,2′-azino-bis-3-ethylbenzthiazoline-6-sulphonic acid (ABTS) and 1,1-diphenyl-2-picrylhydrazyl (DPPH) radicals were used to determine the antioxidant activity of Chromolaena odorata extracts. The Chromolaena odorata crude extract sample was prepared at six concentrations, 0.5, 0.25, 0.125, 0.625 and 0.3125 mg/mL. Then, the antioxidant result of Chromolaena odorata extracts was benchmarked with the gallic acid standard in the concentration range of 2.5–0.1953 µg/mL.

#### 4.5.1. ABTS Assay

The ABTS radical-scavenging test is widely used to determine the antioxidant activity of both hydrophilic and lipophilic compounds. The experiment was performed according to an improved method as described by Heng et al. [46] with some modification. An ABTS solution was prepared at a concentration of 7 mM, then it was kept in darkness at room temperature (25 °C) for 18 h. The *Chromolaena odorata* extracts with different concentrations (0.3125–1 mg/mL) were tested by mixing 160 µL of 7 mM ABTS solution in 96-well microplates. The final absorbance was measured at 734 nm. Then, ABTS radical scavenging activity was calculated as presented in Equation (1),

(1) $$\mathrm{ABTS}\;\mathrm{radical}\;\mathrm{scavenging}\;\mathrm{activity}=\frac{Abs\;control-Abs\;sample\;}{Abs\;control}\times \;100$$

where, *Abs control* is absorbance of ABTS radical and methanol; *Abs sample* is absorbance of ABTS radical and *Chromolaena odorata* extract. All experiments were performed in triplicate.

#### 4.5.2. DPPH Assay

Determination of scavenging stable DPPH free radicals was a very fast method to evaluate the antioxidant activity of the extracts in a very short time. The mechanism of the DPPH method is the determination of the antiradical power of an antioxidant by measurement of the decrease in the absorbance of DPPH at 517 nm. This results in a color change from purple to yellow due to the donation of hydrogen to form a stable DPPH-H molecule. In this study, the DPPH assay method was modified from Omokhua et al. [47] as reported before. The extract was tested at six different concentrations: 0.5, 0.25, 0.125, 0.625, and 0.3125 mg/mL. The DPPH solution was prepared in the concentration of 60 µM, then 40 µL of a methanolic solution of the extract was added. The mixture was shaken vigorously and allowed to stand for 45 min in the dark. The decrease in absorbance was measured at 517 nm against a blank (ethanol) with a spectrophotometer. From a calibration curve obtained with different amounts of extract, the DPPH radical scavenging activity was calculated as described in Equation (2),

(2) $$\mathrm{DPPH}\;\mathrm{radical}\;\mathrm{scavenging}\;\mathrm{activity}=\frac{Abs\;control-Abs\;sample\;}{Abs\;control}\times \;100$$

where, *Abs control* is absorbance of DPPH radical and methanol; *Abs sample* is absorbance of DPPH radical and *Chromolaena odorata* extract. All experiments were performed in triplicate.

### 4.6. Blood Coagulation Test of Chromolaena odorata Extract

Blood coagulation activity of *Chromolaena odorata* extract was evaluated to simulate the function of the extracts in wound hemostatic [48]. In the test, platelets of cow blood were collected by centrifugation of plasma at 400 rpm for 10 min at room temperature and the supernatant fluid was removed. The precipitate was resuspended in a 0.85% NaCl solution (normal saline) and centrifuged again at 400 rpm for 10 min at room temperature. Then, the supernatant fluid was removed. The cleansing processes with normal saline and centrifugation were repeated until the supernatant fluid was clarified. After that, the platelets were stored at 4 °C until further analysis for no longer than 5 days. For the blood coagulation test, the blood sample was diluted in normal saline (1:10 dilution). *Chromolaena odorata* extract at the concentration of 0.0625–1 mg/mL was mixed in a ratio of 1:1 (*v*/*v*). DMSO at a concentration of 1% and normal saline were used as the positive and negative controls, respectively. The blood coagulation was observed using an optical microscope (Eclipse E200MV R, Nikon, Japan).

### 4.7. Cytotoxicity

Cell viability assays were performed based on the 3-(4,5-dimethylthiazol-2-yl)-2,5-diphenyltetrazolium bromide (MTT) colorimetric method. MRC-5 cells which are human diploid cell line were seeded at a density of 10,000 cells per well in a 96-well plate and incubated for 24 h at 37 °C in a 5% CO_2_ atmosphere. Cells were treated with 15.625, 31.25, 62.5, 125, 250, 500 and 1000 μg/mL of *Chromolaena odorata* extract in triplicate for 24 h. Then, 10 µL of 5 mg/mL MTT in PBS solution was directly added into each well and incubated at 37 °C for 3 h. Cultured media was aspirated and a 100 µL aliquot of absolute DMSO was then added. Absorbance (OD) values were measured by a microplate reader (Metertech, Taiwan) at a wavelength of 570 nanometers. The percentage of cell viability was calculated by using the following equation.

(3) % Cell viability=(OD sample−OD blank)/(OD control−OD blank)×100

### 4.8. Formulation of Liquid Plaster

Liquid plaster was prepared by dissolving Plastoid^®^ B together with other excipients in the solvents. The effect of polymer concentration, solvent systems, and amount of plasticizer were varied to find a suitable formulation for further development by loading of *Chromolaena odorata* extract as presented in Table 4. Two reference products of liquid plaster which are available in the market as and OTC products in Thailand were also assessed for comparison purposes.

### 4.9. Evaluation of Liquid Plaster

#### 4.9.1. Rheological Properties

Rheological property is a crucial parameter that needs to be monitored to predict the behavior of the liquid plaster during product application on the skin. Moreover, physical appearances including clarity, precipitation, and viscosity were visually observed and photographed. Then, the rheological behaviors of the liquid plasters were monitored using the rheometer (KINEXUS Lab+, MS603S/01, Malvern Panalytical Ltd., Surrey, UK) in flow curve measurement mode (viscosity vs. shear rate). The measurement temperature was set to 25 °C, the shear rate was set to 0.1–10 Pa.s, and the viscosity at 1 s^−1^ shear rate was chosen as a representative of the formulation. The experiment was carried out in triplicate.

#### 4.9.2. Liquid Drying Time

The drying times of liquid plaster from different formulations were assessed to simulate the practical application of the products. The test was conducted by dropping liquid plaster for 0.5 g on the glass plate. Then the weight change was closely monitored by the 4 digit analytical balance (MS603S/01, METTLER-TOLEDO, Switzerland) for 60 min. Each test was carried out in triplicate.

#### 4.9.3. Film Mechanical Properties

The film sample for the mechanical test was prepared by pouring liquid plaster on the glass plate at the size of 5 × 15 cm^2^. The film was dried under ambient conditions then cut into a size of 1 × 10 cm^2^. The thickness was controlled and measured by the digital caliper. Breaking strain of the testing films was assessed using the texture analyzer (TA.XT.Plus, Stable Micro Systems Ltd., Surrey, United Kingdom). The breaking strain percentage and Young’s modulus were calculated following Equations (4) and (5), respectively.

(4) $$\mathrm{Breaking}\;\mathrm{strain}\left(\%\right)=\frac{Extended\;film\;length-Original\;film\;length}{Original\;film\;length}\times 100$$

(5) $$\mathrm{Young}’s\;\mathrm{modulus}=\frac{\;tensile\;stress}{axial\;strain}\times 100$$

#### 4.9.4. Water Vapor Transmission

Permeability of water vapor is the principal property of the wound dressing patch. Keeping the wound at optimal moisture can promote wound healing via wound re-epithelialization and contraction enhancement [49]. Therefore, water vapor transmission of the thin film from liquid plaster was examined. The film was prepared as described in the film mechanical properties test. Then, the film was then cut into a disc and placed in the mouth of a cylindrical cup filled with 15 g of calcium chloride. The bottle was kept at a temperature of 25–30 °C and 75% ± 5% relative humidity. The weight changes were monitored for 5 days. Then the water vapor transmission rate (WVTR) was calculated as presented Equation (6):

(6) $$\mathrm{Water}\;\mathrm{vapor}\;\mathrm{transmission}\;\mathrm{rate}\;\left(\mathrm{WVTR}\right)=\frac{W_{1}-W_{2}}{t_{2}-t_{1}}$$

where *W*_1_ and *W*_2_ are the initial and endpoint weight of calcium chloride. And *t*_1_ and *t*_2_ is the initial time and the end test time, respectively. All measurements were repeated three times.

#### 4.9.5. Antibacterial Activity of Films Loaded *Chromolaena odorata* Extract

Agar well diffusion method was applied to test the antibacterial activity of films loaded with *Chromolaena odorata* extract. Testing bacteria, *Staphylococcus aureus,* and *Staphylococcus epidermidis,* were prepared at a concentration of 10^8^ CFU/mL which turbidity was equal to a 0.5 McFarland standard. Then, the testing bacterial solutions were spread on the MHA plates. After that, the MHA plates were punctured to receive a well with a diameter size of 8 mm. The liquid plaster loaded with *Chromolaena odorata* extract at concentrations of 0 (negative control), 1, 1.5, and 2 mg/mL, and 25 mg/mL clindamycin solution (positive control) were transferred to the wells on the MHA plates. Then, the plates were incubated at 37 °C for 16–18 h [45].

### 4.10. In Vivo Study of Skin Irritation, Transepidermal Water Loss and Skin Density after Liquid Plaster Use

The in vivo study of skin irritation, transepidermal water loss (TEWL) and skin density after use of the liquid plaster from two optimal formulations and the market reference product A were performed by 27 healthy volunteers ranging in age from 20 to 50 years. The selection of volunteers and test methods were conducted in accordance with the ethical principles described in the Declaration of Burapha University (Ethical code: IRB1-053/2564). The tests on people have to conform to the World Medical Association Declaration of Helsinki [50]. To conduct the study, 0.5 mL of sample was applied to the volunteer’s skin inside part of the arm area for a size of 1 × 3 cm^2^. The film was peeled off after it had been formed (dry film) for 2 min. The 3D imaging camera from Antera 3D (Miravex, Ireland), the skin color probe, TEWL probe, and the high frequency ultrasound probe from DermaLab Combo (Cortex, Denmark) were used to monitor skin parameters such as skin redness for the irritation test, TEWL, and skin density before and after the liquid plaster application.

### 4.11. Statistical Analysis

The data were statistically analyzed using one-way ANOVA followed by Tukey’s Honestly Significant Difference (HSD) post hoc test was used to analyze the data from the three independent experiments.

## Figures and Tables

**Table 1:** Amount and percent yield of gallic acid, quercetin, and apigenin from *Chromolaena odorata* extracts.

Compound	Amount of the Compound (mg)	Percent Yield of the Compound from Extract (%)
Gallic acid	6.59 ± 0.03	0.077 ± 0.000
Quercetin	5.02 ± 0.11	0.059 ± 0.001
Apigenin	17.92 ± 3.53	0.209 ± 0.041

**Table 2:** MIC and MBC of *Chromolaena odorata* extracts against *S. epidermidis* and *S. aureus.*

Pathogenic Bacteria	MIC (mg/mL)	MBC (mg/mL)
*S. epidermidis*	0.25 mg/mL	0.5 mg/mL
*S. aureus*	0.25 mg/mL	0.5 mg/mL

**Table 3:** Shear viscosity, time to dry, and Young’s modulus of blank liquid plaster formulation and liquid plaster formulation-loaded *Chromolaena odorata* extract comparing to the reference products.

	Shear Viscosity at 1 s^−1^ (Pa s)	W_10min_ (%)	Young’s Modulus (MPa)
Blank liquid plaster formulation	9.82	83.09	0.00076
Liquid plaster formulation-loaded *Chromolaena odorata* extract	10.79	80.84	0.00165
Reference product A	2.65	84.47	0.00028
Reference product B	4.49	69.64	0.00046

**Table 4:** Liquid plaster formulations.

Formulation	Ingredients (%*w*/*w*)			
	Plastoid^®^ B	PEG 400	Ethyl Acetate	Isopropyl Alcohol
F 1.1	5	-	95	-
F 1.2	10	-	90	-
F 1.3	15	-	85	-
F 1.4	20	-	80	-
F 1.5	25	-	75	-
F 1.6	30	-	70	-
F 1.7	35	-	65	-
F 2.1	30	-	56	14
F 2.2	30	-	42	28
F 2.3	30	-	28	42
F 2.4	30	-	14	56
F 3.1	30	2	68	-
F 3.2	30	2	55	13
F 3.3	30	2	41	27
F 3.4	30	2	27	41
F 3.5	30	2	13	55
F 4.1	30	5	65	-
F 4.2	30	5	53.5	11.5
F 4.3	30	5	39.5	25.5
F 4.4	30	5	25.5	39.5
F 4.5	30	5	11.5	53.5

## Supplementary Materials

The following are available online at https://www.mdpi.com/article/10.3390/gels8020072/s1, Figure S1: Example images and redness score results obtained from the Antera 3D.
