# Supplementary material for: Development and Evaluation of Liquid Plaster Loaded with Chromolaena odorata Leaf Extract Endowed with Several Beneficial Properties to Wound Healing
Source: Gels. 2022 Jan 24;8(2):72. doi: 10.3390/gels8020072 (PMC8871034; doi:10.3390/gels8020072)
Supplement: Supplementary file 1 [file gels-08-00072-s001.zip › gels-1508873-supplementary.pdf]

Supplementary Materials

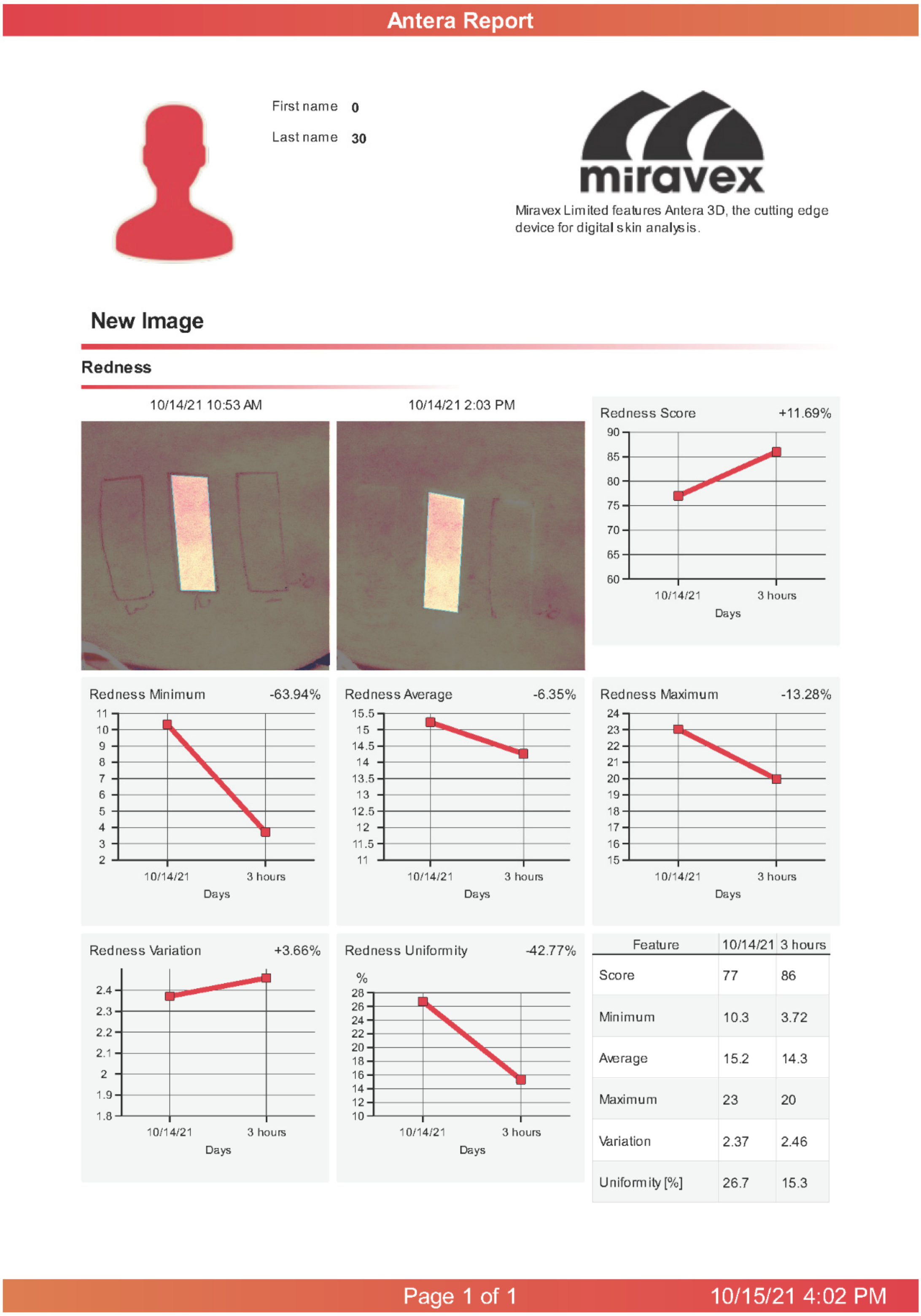

Feature	10/14/21	3 hours
Score	77	86
Minimum	10.3	3.72
Average	15.2	14.3
Maximum	23	20
Variation	2.37	2.46
Uniformity [%]	26.7	15.3

Figure S1. Example images and redness score results obtained from the Antera 3D.
